# Hypernatremia at a Tertiary Hospital Intensive Care Unit in South Africa

**DOI:** 10.7759/cureus.22648

**Published:** 2022-02-27

**Authors:** Limbani Mapata, Guy A Richards, Abdullah E Laher

**Affiliations:** 1 Emergency Medicine, University of the Witwatersrand, Johannesburg, Gauteng, ZAF; 2 Critical Care, University of the Witwatersrand, Johannesburg, Gauteng, ZAF

**Keywords:** outcomes, mortality, sodium, pre-admission hypernatremia, icu-acquired hypernatremia, hypernatremia

## Abstract

Background

Hypernatremia in the critical care setting is a major cause of morbidity and mortality. However, data pertaining to this has not been evaluated in South African hospitals. The aim of this study was to evaluate hypernatremia with regards to its prevalence, associated factors, and outcomes at an academic hospital intensive care unit (ICU) in Johannesburg, South Africa.

Methods

The ICU charts of patients admitted to the Charlotte Maxeke Johannesburg Academic Hospital adult general ICU from June 1, 2016 to May 31, 2017 were retrospectively reviewed. Subjects were categorized into three groups namely, ICU-acquired hypernatremia (IAH), pre-admission hypernatremia (PAH), and normonatremia. Data was compared between the three groups.

Results

Of the 833 subjects that were enrolled, 310 (37.2%) were hypernatremic. IAH was present in 144 (17.2%) and PAH in 166 (19.9%) subjects. Hypernatremia was significantly (p <0.05) associated with a higher rate of altered mental status, higher Acute Physiologic Assessment and Chronic Health Evaluation II (APACHE II) scores, a higher rate and duration of mechanical ventilation, a greater need for inotropic/vasopressor support, longer ICU stay and higher ICU mortality.

Conclusion

Hypernatremia in ICU patients remains a significant contributor to morbidity, mortality, and ICU length of stay. The prevalence of hypernatremia was much higher than that reported in higher-income countries.

## Introduction

Dysnatremia is a significant problem in the critically ill [1]. Despite a halving of the incidence of hyponatremia in recent years, the incidence of hypernatremia has doubled [2-4]. Hypernatremia is predominantly caused by loss of free water and less commonly by sodium (Na) excess [5,6]. Hypernatremia may be present in approximately 6%-42% of patients in the ICU and is in general, associated with a 50%-60% increase in short-term mortality. Even when the degree of hypernatremia is mild (Na <150 mmol/L), the associated 30-day mortality is still more than 20% [7].

Sodium is the most abundant cation in extracellular fluid. The serum Na concentration is crucial for the maintenance of extracellular tonicity and the movement of water across cell membranes [8]. As such, the clinical manifestations of hypernatremia are predominantly due to the osmotic shift of fluid from the intracellular to the extracellular space [5]. The clinical presentation of hypernatremia is dependent on its severity and rapidity of onset and includes thirst, muscle weakness, lethargy, restlessness, irritability, confusion, seizures, and a decreased level of consciousness. In advanced cases, hypernatremia may also lead to stretching and rupture of intracranial blood vessels [9].

In critically ill patients, hypernatremia may be classified as ICU-acquired hypernatremia (IAH) or pre-admission hypernatremia (PAH) [10]. Infants and the elderly account for a large proportion of PAH, while hypernatremia acquired during hospital admission is more prevalent in the intensive care unit (ICU) setting than the general wards [11]. The prevalence of hypernatremia in the ICU setting ranges from 6%-42%, with most cases manifesting within the first week of admission. In contrast, the incidence of hypernatremia acquired prior to ICU admission is much lower at approximately 4% [12,13].

Both IAH and PAH have been shown to be independent predictors of mortality and longer ICU stay [14-16], with the latter more so than the former [17,18]. The most likely cause of hypernatremia acquired during hospital admission is inadequate fluid intake which is exacerbated by ongoing fluid losses. In addition, solute loading from medications and nutrition also contribute to the development of hypernatremia [19]. Several factors, including a high Acute Physiologic Assessment and Chronic Health Evaluation II (APACHE II) score, a low Glasgow Coma Scale (GCS) score, the presence of organ dysfunction and transfusion of blood, and blood products have been reported to be associated with IAH [13].

There is lack of data pertaining to hypernatremia in the ICU setting emanating from South Africa and other developing countries. Hence, the aim of this study was to evaluate hypernatremia with regard to its prevalence, associated factors, and outcomes at an academic hospital ICU in Johannesburg, South Africa.

## Materials and methods

The ICU charts of patients admitted to the Charlotte Maxeke Johannesburg Academic Hospital (CMJAH) adult general ICU from June 1, 2016 to May 31, 2017 were retrospectively reviewed. CMJAH is a tertiary-level academic hospital that is affiliated with the University of the Witwatersrand. The hospital has a capacity of approximately a thousand beds that includes 19 adult multidisciplinary ICU beds. In general, laboratory serum Na and electrolyte concentrations are measured at least once daily in all patients admitted to the ICU.

For the purpose of this study, hypernatremia was defined as the presence of two consecutive laboratory serum Na levels >145 mmol/L. Similarly, hyponatremia was defined as the presence of two consecutive laboratory serum Na levels <135 mmol/L. All other patients were categorized as normonatremic. Hypernatremia was further categorized as mild (serum Na concentration 146-149 mmol/L), moderate (serum Na concentration 150-155 mmol/L) and severe (serum Na concentration >155 mmol/L) [13]. Acute kidney injury (AKI) was defined as either an increase in serum creatinine by ≥26.4 μmol/L within 48 hours or an increase in serum creatinine to ≥1.5 times its baseline within the last seven days or a decrease in urine output of <0.5 mL/kg/h for >6 hours [20].

After obtaining permission to conduct the study and ethics clearance from the hospital manager and the Human Research Ethics Committee of the University of the Witwatersrand (clearance certificate no. M170976), respectively, the ICU charts of all patients admitted to the ICU over the data collection period were retrieved from the medical records department. Patients with hyponatremia as well as those whose medical records could not be found were excluded from the study.

Data collection was conducted by the primary investigator and closely monitored by the study supervisors. The following data were entered into the study data collection sheet: subject age, sex, the presence of an altered mental status during the course of ICU admission, APACHE II score at ICU admission, daily serum Na concentration, severity, duration and time to onset of hypernatremia, presence AKI, requirement for hemodialysis, requirement for mechanical ventilation, requirement for inotropic/vasopressor support, length of ICU stay and ICU mortality. Study subjects were classified into three categories IAH, PAH, and normonatremia. To maintain patient anonymity and confidentiality, patient identifying information was not captured. Furthermore, study data was stored in a password-protected computer that was only accessible to the study investigators.

Collected data was thereafter entered into Microsoft® Excel® (Microsoft 365, Version 16.0.13029.20232) and subsequently exported to Stata version 16 (StataCorp Limited, Texas, United States of America) for statistical analysis. Data was described and compared between the three groups (IAH, PAH, and normonatremia). Categorical variables were described using frequency and percentage and compared by means of the Pearson’s chi-square test. Since the Shapiro-Wilk test indicated that all the categories of continuous data were not normally distributed, these were described using the median and interquartile range (IQR) and compared by means of the Kruskal-Wallace test. The odds ratio (OR) and 95% confidence interval (CI) were used to compare ICU mortality between those with mild, moderate, and severe hypernatremia. The level of significance was set at p = 0.05. Study reporting conformed with STROBE (Strengthening the Reporting of Observational Studies in Epidemiology) guidelines [21].

## Results

As per the ICU register, 991 patients were admitted to the ICU over the data collection period. Of these, 126 patients developed hyponatremia while the medical records of 32 could not be found. Hence, a total of 833 patients were included in the final study sample. Of these, 523 (62.8%) subjects were categorized as normonatremia and 310 (37.2%) as hypernatremia. Of the 310 subjects with hypernatremia, 144 (17.2%) were categorized as IAH and 166 (19.9%) as PAH.

Table 1 describes and compares the various characteristics of the study population. The median age of study subjects was 45 (IQR 31-61) years. There was a slightly higher proportion of female subjects (n=457, 54.9%). There were no significant differences between the three groups with regards to age, sex, the median duration of hypernatremia during ICU stay, and requirement for hemodialysis (p >0.05). Mild hypernatremia was significantly more prevalent in subjects with IAH, whereas moderate and severe hypernatremia were significantly more prevalent in subjects with PAH (p <0.05).

**Table 1:** Comparison of characteristics between the three groups of study subjects AKI – acute kidney injury, IAH – ICU-acquired hypernatremia, PAH – pre-admission hypernatremia, NN – normonatremia, Na – sodium ^a ^Only includes the duration of hypernatremia during hospital admission and does not include the duration of hypernatremia if the onset was prior to hospital admission. ^b^ *IAH vs PAH, p =0.007; IAH vs NN, p <0.001; PAH vs NN, p <0.001* ^c^ *IAH vs PAH, p =0.004; IAH vs NN, p =0.001; PAH vs NN, p <0.001* ^d^ IAH vs PAH, p =0.309; IAH vs NN, p =0.287; *PAH vs NN, p =0.015* ^e^ *IAH vs PAH, p =0.002; IAH vs NN, p <0.001; PAH vs NN, p <0.001* ^f^ *IAH vs PAH, p <0.001; IAH vs NN, p <0.001; PAH vs NN, p <0.001* ^g^ IAH vs PAH, p =0.289; I*AH vs NN, p <0.001; PAH vs NN, p =0.002* ^h^ *IAH vs PAH, p <0.001; IAH vs NN, p <0.001; PAH vs NN, p <0.001* ^i ^IAH vs PAH, p =0.443; *IAH vs NN, p <0.001; PAH vs NN, p <0.001* Italics denotes statistically significant differences between groups

Characteristics	Entire Cohort (n=833)	ICU-acquired hypernatremia (n=144)	Pre-admission hypernatremia (n=166)	Normonatremia (n=523)	P-value
Age (median, IQR)	45 (31-61)	40 (31-57)	46 (33-62)	46 (31-62)	p=0.186
Sex					p=0.423
Female (n, %)	457 (54.9)	86 (59.7)	88 (53.0)	283 (54.1)	
Male (n, %)	376 (45.1)	58 (40.3)	78 (47.0)	240 (45.9)	
Altered mental status (n, %)	308 (37.0)	71 (49.3)	107 (64.5)	130 (24.9)	^b^p<0.001
APACHE II score (median, IQR)	14 (9-20)	15 (10-21)	17 (12-25)	12 (8-19)	^c^p<0.001
Severity of hypernatremia					
Mild (Na 146-149 mmol/L) (n, %)	192 (20.0)	105 (72.9)	87 (52.4)	NA	p<0.001
Moderate (Na 150-154 mmol/L) (n, %)	86 (9.0)	31 (21.5)	55 (33.1)	NA	p=0.023
Severe (Na ≥155 mmol/L) (n, %)	32 (3.3)	8 (5.6)	24 (14.5)	NA	p=0.010
Time to onset of hypernatremia (days) (median, IQR)	NA	3 (2 – 4)	NA	NA	NA
^a^Duration of hypernatremia (days) (median, IQR)	NA	3 (2 – 5)	3 (2 – 6)	NA	p=0.617
AKI (n, %)	374 (44.9)	68 (47.2)	88 (53.0)	221 (42.3)	^d^p=0.046
Required hemodialysis (n, %)	192 (23.0)	34 (23.6)	45 (27.1)	113 (21.6)	p=0.336
Required mechanical ventilation (n, %)	540 (64.8)	133 (92.4)	133 (80.1)	274 (52.4)	^e^p<0.001
Duration of mechanical ventilation (days) (median, IQR)	1 (0-3)	4 (3 – 7)	2 (1 – 5)	1 (0 – 2)	^f^p<0.001
Required inotropic/vasopressor support (n, %)	309 (37.1)	72 (50.0)	73 (44.0)	164 (31.4)	^g^p<0.001
Length of ICU stay (days) (median, IQR)	2 (1 – 5)	7 (4 – 11)	4 (2 – 8)	2 (1 – 3)	^h^p<0.001
ICU mortality (n, %)	214 (25.7)	52 (36.1)	67 (40.4)	95 (18.1)	^i^p<0.001

When comparing the IAH and PAH groups, requirement for mechanical ventilation, the median duration of mechanical ventilation, and the median length of ICU stay were significantly higher among those with IAH (p <0.05), while altered mental status and the median APACHE II score were significantly higher in the PAH group (p <0.05). There were no mortality differences between the two groups (p >0.05)

When comparing the IAH and the normonatremia groups, altered mental status, the median APACHE II score, requirement for mechanical ventilation, the median duration of mechanical ventilation, requirement for inotropic/vasopressor support, the median length of ICU stay, and ICU mortality were significantly higher in the IAH group (p <0.05).

When comparing the PAH and the normonatremia groups, altered mental status, the median APACHE II score, the presence of AKI, requirement for mechanical ventilation, the median duration of mechanical ventilation, requirement for inotropic/vasopressor support, the median length of ICU stay and ICU mortality were significantly higher in the PAH group (p <0.05).

Table 2 compares ICU mortality in relation to the severity of hypernatremia. Compared to subjects with normonatremia, there was an incremental increase in the likelihood (odds ratio) of ICU mortality with an increase in the severity of hypernatremia. The likelihood of ICU mortality was 5.79 times higher among those with severe hypernatremia (p <0.001) compared to those with normonatremia.

**Table 2:** Comparison of ICU mortality with the severity of hypernatremia

Degree of dysnatremia	Total (n=833)	ICU mortality (n=214)	Survival to ICU discharge (n=619)	OR (95% CI)	p-value
Normonatremia (Na 135-145 mmol/L) (n, %)	523 (62.8)	95 (44.4)	428 (69.1)	1.00 (Reference)	
Mild hypernatremia (Na 146-149 mmol/L) (n, %)	192 (23.0)	63 (29.4)	129 (20.8)	2.20 (1.51-3.20)	<0.001
Moderate hypernatremia (Na 150-154 mmol/L) (n, %)	86 (10.4)	38 (17.8)	48 (7.8)	3.57 (2.21-5.76)	<0.001
Severe hypernatremia (Na ≥155 mmol/L) (n, %)	32 (3.8)	18 (8.4)	14 (2.3)	5.79 (2.78-12.06)	<0.001

## Discussion

In this study, the prevalence of hypernatremia was 37.2%, with mild, moderate, and severe hypernatremia accounting for 20.0%, 9.0%, and 3.3% of the total number of cases respectively. Of note, hypernatremia was significantly (p <0.05) associated with a higher rate of altered mental status, higher APACHE II scores, a higher rate and duration of mechanical ventilation, a greater need for inotropic/vasopressor support, longer ICU stay, and higher ICU mortality. These variables all indicate that hypernatremia is associated with a greater severity of illness and worse outcomes in ICU patients. When comparing the IAH and PAH groups, the rate of altered mental status and the median APACHE II scores were significantly (p <0.05) higher in the PAH group, while requirement for mechanical ventilation and longer ICU stay were significantly (p <0.05) greater in the IAH group. There were however no significant (p >0.05) mortality differences between the two groups. This indicates that hypernatremia in general, irrespective of etiology, is associated with poorer outcomes in the ICU.

Compared to the findings of this study, a multicenter study that analyzed the data of 151 486 adult patients from 77 ICUs in Austria, reported the prevalence of hypernatremia to be much lower at 6.9%, with mild, moderate, and severe hypernatremia accounting for 5.1%, 1.2% and 0.6% of the total number of cases respectively. The likelihood of mortality for mild, moderate, and severe hypernatremia was 1.48 (1.36-1.61), 2.32 (1.98-2.73), and 3.64 (2.88-4.61) times higher, which is lower than the findings of our study [2.20 (1.51-3.20); 3.57 (2.21-5.76) and 5.79 (2.78-12.06) respectively]. The authors also reported a higher mean age of 62 years and a higher proportion of male patients in their cohort. Similar to our findings, the authors also reported a longer length of ICU stay (5 versus 3 days; p <0.001) and higher mortality (21.1%-46.1% versus 9.7%; p <0.001) in patients with hypernatremia compared to those with normonatremia [12].

Another multicenter study that analyzed the medical records of 8 140 patients from 12 ICUs in France reported that the prevalence of IAH was 15.3%. The authors of this study classified the degree of hypernatremia as mild (serum Na 146-150 mmol/L) and moderate to severe (serum Na >150 mmol/L). Male gender, illness severity at ICU admission, and the presence of organ failure or requirement for life-supporting medication at ICU admission were independently associated with hypernatremia. Both mild, as well as moderate to severe IAH, were independently associated with mortality (sub-distribution hazard ratio (SHR) of 2.03 and 2.67 respectively) [22]. A recent study that included the data of ICU patients from 208 hospitals in the USA, reported that among 88 160 patients that were admitted to the ICU with normonatremia, approximately 7% developed IAH. Compared to those with normonatremia, the IAH group was older, had higher illness severity scores, had a longer length of ICU stay, and had higher mortality (p <0.05) [23].

In a study that was prospectively conducted at an ICU in Sri Lanka, 74 of 174 (42.5%) patients developed IAH. Compared to patients with normonatremia, those with IAH had a higher APACHE II score, a lower GCS score, a higher prevalence of organ dysfunction, and required more blood and blood product transfusions. Additionally, patients with IAH had a much longer mean length of ICU stay (11 days vs 4.8 days, p< 0.001) and a much higher rate of ICU mortality (43% vs 15%, p<0.001) [13]. Contrary to the findings of our study where there were no significant differences in mortality between the IAH and PAH groups, a study in the Netherlands reported that patients with IAH had a significantly higher mortality than those with PAH [18].

A recently published study that was conducted in Cape Town, South Africa reported that the prevalence of hypernatremia among total hospital admissions over a three-month period was 1.5%. Among those with hypernatremia, 32.4% had hypernatremia on admission, 67.6% developed hypernatremia during the course of hospital admission, 49.5% were male, the mean age was 53 years, 24.0% were managed in the ICU and the in-hospital mortality rate was 38.7%. Mortality was higher among patients with more severe hypernatremia [24]. Since the study comprised of all hospitalized patients and not just patients that were admitted to the ICU, this is the most likely reason that the prevalence of hypernatremia was much lower compared to the findings of our study.

The relatively high prevalence of hypernatremia in our study is concerning. Contrary to the common understanding regarding the etiology of IAH, a previous study concluded that inadequate cation exchange (renal sodium handling) due to severe illness, rather than Na overload or water deficit was mostly responsible for the development of IAH [25]. Although we did not collect data pertaining to daily Na intake, the APACHE II score were significantly higher in subjects with hypernatremia compared to those with normonatremia indicating that subjects with hypernatremia presented with more severe illness. Furthermore, since the prevalence of hypernatremia reported in our study as well as the study conducted in Sri Lanka was much higher compared to that reported in higher-income countries (see above references), this may be in keeping with the fact that ICU patients in lower-income countries have an overall higher severity of illness compared to ICU patients in higher-income countries [26]. There is a need to further explore this observation in future studies.

Interventions should be aimed at reducing the prevalence of hypernatremia in the ICU by instituting early management of the underlying illness and thereby preventing progression to severe illness, coupled with meticulous attention to Na intake and fluid administration. There is a need to conduct similar designed studies in other low-middle-income settings. Future studies should also aim to define the role of Na overload/water deficit versus the role of illness severity on the development of hypernatremia in the ICU setting.

There are several limitations to this study. Firstly, this was a single-center study, hence the findings of this study may not reflect those at other facilities in South Africa. Secondly, the study did not determine the total number of days with hypernatremia and correlate it with outcomes. Thirdly, we did not determine the role of Na intake and fluid deficit on the development of hypernatremia and its effect on patient outcomes. Fourthly, among subjects that were categorized as PAH, we did not distinguish between hypernatremia that was present prior to hospital admission and hypernatremia that developed in the general ward prior to ICU admission.

## Conclusions

The prevalence of hypernatremia in this study was 37.2%, which is much high than that reported in higher-income countries. Hypernatremia was significantly associated with a higher rate of altered mental status, higher APACHE II scores, a higher rate and duration of mechanical ventilation, a greater need for inotropic/vasopressor support, longer ICU stay, and higher ICU mortality. Overall, increasing severity of hypernatremia was associated with significantly higher mortality. Future studies should also aim to define the role of Na overload/water deficit versus the role of illness severity on the development of hypernatremia in the ICU setting.
